# Intraplantar β-Caryophyllene Alleviates Pain and Inflammation in STZ-Induced Diabetic Peripheral Neuropathy via CB_2_ Receptor Activation

**DOI:** 10.3390/ijms26094430

**Published:** 2025-05-07

**Authors:** Amina M. Bagher

**Affiliations:** Department of Pharmacology and Toxicology, Faculty of Pharmacy, King Abdulaziz University, Jeddah 21589, Saudi Arabia; abagher@kau.edu.sa; Tel.: +966-547187973

**Keywords:** β-Caryophyllene, diabetic peripheral neuropathy, cannabinoid receptors 2, STZ-induced neuropathy

## Abstract

Diabetic peripheral neuropathy (DPN) is a debilitating complication of diabetes, characterized by mechanical allodynia, neuroinflammation, and oxidative stress. Current treatments offer limited efficacy and are often associated with systemic side effects. Emerging evidence suggests that activation of cannabinoid receptor type 2 (CB_2_) may represent a promising target for managing neuropathic pain and inflammation. This study investigates the therapeutic potential of intraplantar β-Caryophyllene (BCP), a selective CB_2_ receptor agonist, administered as a topical intervention in a streptozotocin (STZ)-induced DPN mouse model. Hyperglycemia was induced by STZ injections, and diabetic mice received intraplantar BCP (9, 18, or 27 µg) daily for 21 days. Mechanical allodynia was assessed using von Frey filaments, and levels of pro-inflammatory cytokines (TNF-α, IL-1β, IL-6) and oxidative stress markers (MDA, SOD, CAT) were quantified in hind paw tissues. BCP dose-dependently alleviated STZ-induced mechanical allodynia, with the 27 µg dose producing the most pronounced effect (*p* < 0.001). The anti-allodynic effects of BCP were mediated through CB_2_ receptor activation, confirmed by reversal with the CB_2_ antagonist AM630 (*p* < 0.001), while the CB_1_ antagonist AM251 had no significant impact. In addition, BCP significantly reduced pro-inflammatory cytokines (*p* < 0.01) and oxidative stress markers (*p* < 0.001) while restoring antioxidant enzyme activities (*p* < 0.05). A control group treated with a clinically available topical analgesic cream containing capsaicin 0.075% exhibited limited efficacy. These findings position topical BCP administration as a novel therapeutic strategy for DPN, offering sustained pain relief and modulation of neuroinflammatory and oxidative pathways with minimal systemic exposure. Further clinical studies are warranted to validate its potential for translation into therapeutic practice.

## 1. Introduction

Diabetic peripheral neuropathy (DPN) is one of the most common and debilitating complications of diabetes, affecting nearly 50% of diabetic individuals and leading to progressive degeneration of peripheral nerves, primarily in the lower extremities [1,2,3]. A 2022 systematic review and meta-analysis estimated the prevalence of DPN in Saudi Arabia to be approximately 39%, highlighting its significant burden in the region [4]. Clinically, DPN typically presents in its early stages with chronic spontaneous pain, mechanical allodynia, and thermal hyperalgesia, predominantly affecting the feet and hands. Over time, the degeneration of tiny sensory fibers can result in hypoalgesia, reducing pain perception and significantly increasing the risk of serious complications, such as diabetic foot ulcers, infections, and lower limb amputations [2,5,6].

The pathogenesis of DPN is complex and multifactorial, involving metabolic disturbances, ischemic injury, and hyperglycemia-induced inflammation, which collectively drive nociceptor sensitization and neuronal dysfunction [7,8]. Among these mechanisms, inflammation plays a pivotal role. Chronic hyperglycemia elevates pro-inflammatory cytokines, including tumor necrosis factor-alpha (TNF-α), interleukin-1β (IL-1β), and interleukin-6 (IL-6), which exacerbate nerve damage by promoting oxidative stress, nociceptor sensitization, and neuroinflammation. Hyperglycemia also increases the production of reactive oxygen species (ROS), overwhelming the antioxidant defense system and contributing to oxidative injury in peripheral nerves. This ROS-mediated damage further amplifies inflammatory signaling, activating pathways such as NF-κB, which perpetuate inflammation and impair nerve repair processes [7,8]. These interconnected mechanisms underscore the importance of targeting both inflammation and oxidative stress in the therapeutic landscape of DPN.

Current treatments for DPN focus primarily on symptom management rather than disease modification. Pharmacological agents, such as gabapentin and pregabalin, duloxetine and amitriptyline, and opioids like tramadol, are widely used for neuropathic pain relief. However, their clinical utility is limited by substantial side effects, including dizziness, sedation, and gastrointestinal disturbances, which contribute to poor adherence and insufficient pain control [2,9,10]. Topical treatments, such as lidocaine 5% patches and capsaicin—available as 8% patches and 0.075% cream—provide localized pain relief with fewer systemic side effects, provide localized pain relief with fewer systemic side effects but are effective in less than half of patients and fail to address the underlying pathophysiological changes in DPN [11]. These limitations emphasize the urgent need for novel therapeutic approaches that not only alleviate pain symptoms but also target the root causes of DPN, particularly neuroinflammation and oxidative stress.

One candidate compound gaining significant attention in neuropathic pain research is β-Caryophyllene (BCP), a natural sesquiterpene found in essential oils derived from various plants, including clove, black pepper, rosemary, and *Cannabis sativa* [12,13]. BCP is widely recognized for its anti-inflammatory, antioxidant, and anti-nociceptive properties, making it a compelling candidate for treating inflammatory and neuropathic pain conditions [14,15,16,17,18,19,20]. Mechanistically, BCP selectively activates the cannabinoid receptor type 2 (CB_2_), a G-protein coupled receptor (GPCR) predominantly expressed on immune cells and nociceptive neurons. Unlike cannabinoid receptor type 1 (CB_1_), CB_2_ receptor activation regulates immune responses and pain signaling without inducing psychoactive side effects [21,22,23].

Preclinical studies have consistently shown that systemic administration of BCP effectively alleviates neuropathic pain and inflammation in rodent models, including those of sciatic nerve injury, chemotherapy-induced neuropathy, and diabetes-induced neuropathy. BCP’s therapeutic efficacy is primarily attributed to its ability to suppress pro-inflammatory cytokines such as TNF-α, IL-1β, and IL-6. It also mitigates oxidative stress by enhancing antioxidant enzyme activity and alleviates key pain symptoms, including mechanical allodynia and thermal hyperalgesia [14,15,19,24]. Additionally, chronic oral administration of BCP in streptozotocin (STZ)-induced diabetic mice not only alleviated neuropathic pain but also reduced depressive-like behavior, a common comorbidity in DPN. These therapeutic effects were associated with decreased substance P and pro-inflammatory cytokines, reinforcing BCP’s dual action on pain modulation and inflammatory suppression [25].

Current findings highlight the therapeutic potential of BCP in managing both neuropathic and inflammatory pain. Although systemic BCP administration has demonstrated significant efficacy, topical delivery offers distinct advantages by selectively targeting peripheral receptors while minimizing systemic exposure and associated side effects. Given that DPN predominantly affects distal extremities, localized therapeutic approaches targeting peripheral nociceptive sites may enhance efficacy and safety profiles [23]. Importantly, CB_2_ receptor activation provides a non-psychoactive alternative to conventional cannabinoid-based therapies, further supporting BCP’s clinical appeal [26]. In this study, we evaluated the effectiveness of topical BCP administration via intraplantar (i.pl.) injections in an STZ-induced diabetic mouse model. We hypothesized that topical BCP would activate peripheral CB_2_ receptors, leading to significant pain relief and suppression of inflammatory responses. This proof-of-concept study aims to establish a novel, targeted therapeutic strategy for DPN, potentially improving clinical outcomes. As a clinical comparator, we employed a topical analgesic cream containing capsaicin 0.075%, which is approved for the symptomatic relief of painful DPN.

## 2. Results

### 2.1. Impact of BCP on Body Weight and Blood Glucose Levels in an STZ-Induced DPN Mouse Model

**Figure 1:**
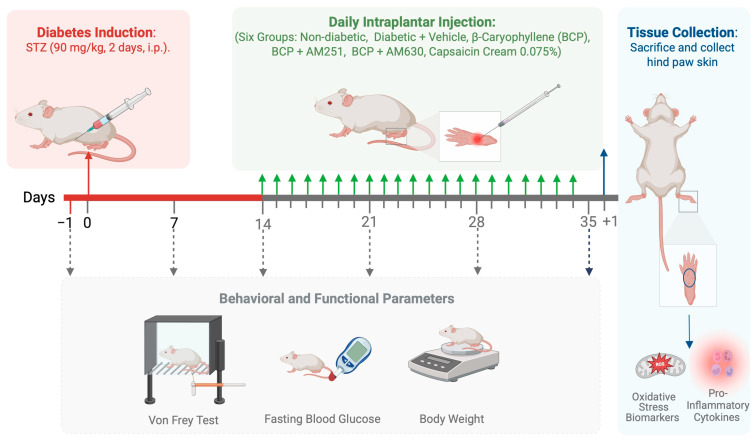
Schematic representation of the experimental timeline. This diagram illustrates the experimental timeline, outlining the procedures for diabetes induction and BCP treatment. Mice were induced with diabetes via STZ injections (90 mg/kg, i.p.). Starting on day 14, daily intraplantar (i.pl.) injections of β-Caryophyllene (BCP, 27 µg) were administered for 21 days to six groups: non-diabetic, diabetic + vehicle, diabetic + BCP, diabetic + BCP + AM251 (CB_1_ antagonist), diabetic + BCP + AM630 (CB_2_ antagonist), and diabetic + topical analgesic cream with capsaicin 0.075% (Zostrix^®^ HP). Behavioral and functional parameters, including mechanical allodynia (assessed by the Von Frey test), fasting blood glucose levels, and body weight, were evaluated at baseline (before STZ injection) and then on days 7, 14, 21, 28, and 35. On day 35, mice were sacrificed, and hind paw skin was collected to analyze oxidative stress biomarkers and pro-inflammatory cytokines. The figure was created with BioRender.com.

**Figure 2:**
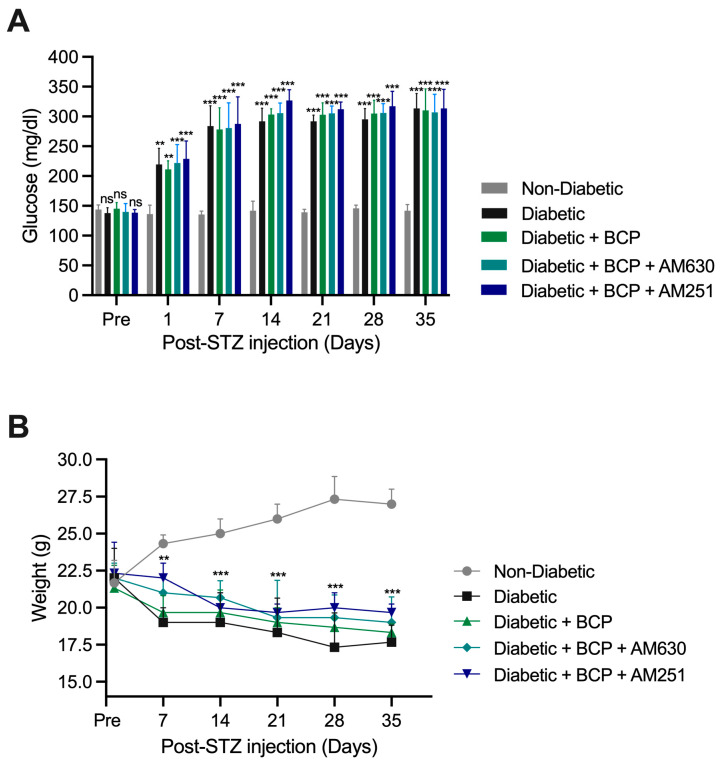
Effects of STZ-induced diabetes and BCP treatment on blood glucose and body weight. (**A**) STZ-induced diabetes resulted in glucose levels exceeding 250 mg/dL. BCP treatment, either alone or with AM251 (CB_1_ antagonist) or AM630 (CB_2_ antagonist), did not significantly alter glucose levels in diabetic mice. Non-diabetic controls maintained normal glucose levels. (**B**) Diabetic mice showed significant weight loss starting on day 7 post-STZ injection, with this reduction persisting throughout the study. BCP and antagonist treatments had no significant impact on body weight. Data are presented as mean ± SEM (*n* = 8 per group). Statistical analysis: Two-way ANOVA with Tukey’s post-hoc test. ns, not significant, ** *p* < 0.01, *** *p* < 0.001 vs. non-diabetic controls.

The experimental timeline is illustrated in Figure 1. To evaluate the systemic effects of BCP, an STZ-induced diabetic peripheral DPN model was used. Diabetes was induced with intraperitoneal (i.p.) injections of STZ (90 mg/kg) on two consecutive days, while non-diabetic controls received normal saline. By day 7 post-STZ injection, diabetes was successfully induced, as evidenced by fasting blood glucose levels exceeding 250 mg/dL, and this diabetic state was maintained throughout the study. Non-diabetic controls maintained normal glucose levels, confirming the absence of diabetes in this group (Figure 2A). BCP treatment via i.pl. injection (27 µg), or pretreatment with AM630 or AM251, did not alter blood glucose levels in diabetic mice compared to untreated diabetic controls (Figure 2A).

Diabetic mice showed a significant reduction in body weight starting on day 7 post-STZ injection (*p* < 0.01) compared to non-diabetic controls, and this weight loss persisted throughout the study period. However, BCP injections, with or without pretreatment with AM630 or AM251, had no significant impact on body weight in diabetic mice (Figure 2B). These findings confirm the successful induction of diabetes in STZ-treated mice and indicate that BCP treatment does not influence key systemic parameters such as blood glucose levels or body weight.

### 2.2. Acute Local BCP Injections Alleviate Mechanical Allodynia in STZ-Induced DPN Mouse Model via Peripheral CB_2_ Receptor Mediation

**Figure 3:**
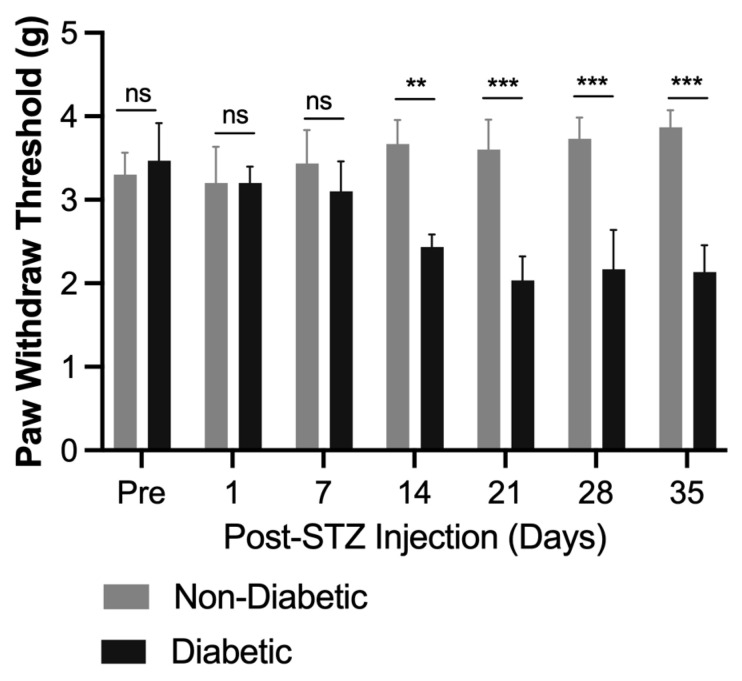
Time course of mechanical withdrawal thresholds in STZ-induced diabetic mice. Mechanical allodynia was assessed using von Frey filaments in STZ-induced diabetic and non-diabetic control mice. Diabetic mice exhibited a significant reduction in paw withdrawal thresholds starting from day 7 post-STZ injection, with the reduction becoming more pronounced by day 14 and persisting through day 35. Non-diabetic controls maintained stable thresholds throughout the study. Thresholds are expressed in grams (g) and represent mean ± SEM (*n* = 8 per group). Statistical analysis: Two-way ANOVA with Bonferroni correction. ns, not significant, ** *p* < 0.01, *** *p* < 0.001 vs. non-diabetic controls.

To determine the onset of mechanical allodynia in the STZ-induced mouse model, mechanical allodynia was assessed using von Frey filaments at baseline and on days 1, 7, 14, 21, 28, and 35 post-STZ injection (Figure 3). Diabetic mice exhibited a significant reduction in paw withdrawal thresholds compared to non-diabetic controls starting on day 7 post-STZ injection (*p* < 0.01), indicating the development of mechanical allodynia. This reduction became more pronounced by day 14 and persisted through day 35. Non-diabetic mice maintained consistent thresholds, confirming the impact of diabetes on nociceptive sensitivity. Based on this timeline, day 14 was chosen as the optimal starting point for BCP treatment, as this time point reflects the peak of mechanical allodynia.

**Figure 4:**
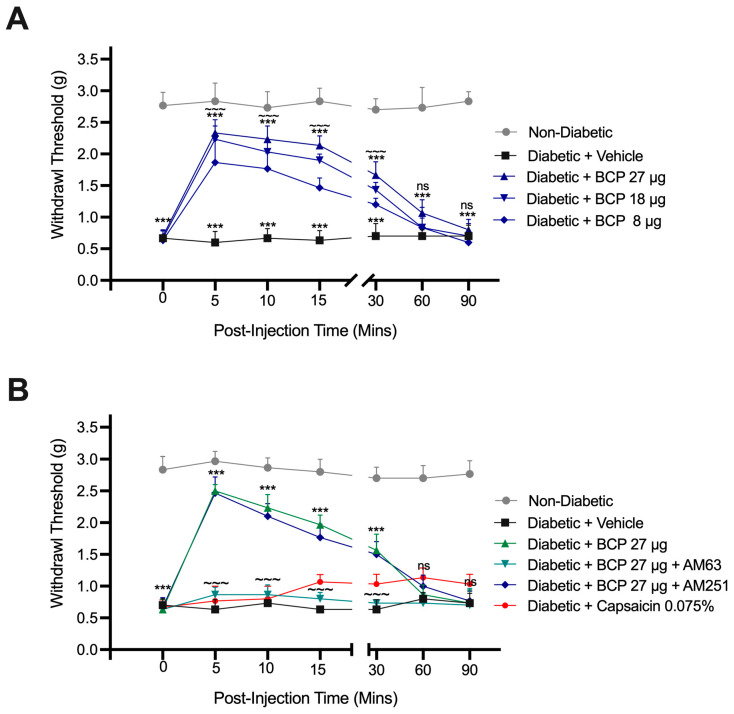
Effects of localized β-Caryophyllene (BCP) injections on mechanical allodynia in diabetic mice. (**A**) Dose-dependent effects of intraplantar (i.pl.) administration of BCP (8, 18, and 27 µg) on paw withdrawal thresholds in diabetic mice. Paw withdrawal thresholds were measured over 90 min post-BCP injection on day 14 after STZ injection. *** *p* < 0.001 compared to non-diabetic controls; ~~~ *p* < 0.001 compared to diabetic controls; ns indicates no significant difference compared to diabetic controls. (**B**) Effects of CB receptor antagonists on BCP (27 µg)-induced alleviation of mechanical allodynia. Diabetic mice were pretreated with AM630 (CB_2_ antagonist) or AM251 (CB_1_ antagonist) 30 min before BCP injection. As a positive control, mice were treated with a topical analgesic cream containing capsaicin 0.075%. Data are presented as mean ± SEM (*n* = 8 per group). Statistical analysis: two-way ANOVA with Tukey’s post-hoc test. ns, not significant, *** *p* < 0.001, compared to diabetic controls; ns indicates no significant difference compared to diabetic controls; ~~~ *p* < 0.001 compared to BCP (27 µg)-treated diabetic mice. Statistical analysis: two-way ANOVA with Tukey’s post-hoc test.

A dose-response analysis of BCP, administered via i.pl. injections at 8, 18, and 27 µg doses on day 14, revealed a dose-dependent alleviation of mechanical allodynia. Paw withdrawal thresholds increased significantly following i.pl. injections of BCP at all tested doses compared to vehicle-treated diabetic controls (*p* < 0.05; Figure 4A). Among the doses, 27 µg BCP produced the most pronounced and sustained effect, with peak efficacy observed 15 min post-injection and a return to baseline by 90 min. Further investigation confirmed the mechanism of action for BCP’s anti-allodynic effects. Pretreatment with the CB_2_ antagonist AM630 significantly reversed the effects of BCP (*p* < 0.001), confirming CB_2_ receptor involvement, while the CB_1_ antagonist AM251 had no significant impact (Figure 4B). These results identify CB_2_ receptor activation as the primary mechanism behind BCP’s rapid and transient anti-allodynic efficacy in DPN.

For comparison, mice treated with a single dose of topical analgesic cream containing capsaicin 0.075% showed no improvement in mechanical allodynia, consistent with prior data suggesting that topical capsaicin application at 0.075% requires prolonged application for efficacy [27]. In conclusion, i.pl. injections of 27 µg BCP provide a rapid and effective onset of action in alleviating mechanical allodynia after a single application, demonstrating superior efficacy compared to topical capsaicin cream (0.075%).

### 2.3. Sustained Anti-Allodynic Effects of Chronic Topical BCP Injections in STZ-Induced DPN Mouse Model

**Figure 5:**
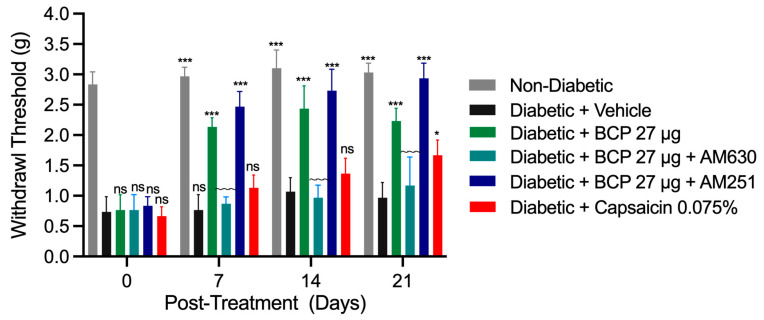
Sustained anti-allodynic effects of chronic topical BCP injections in STZ-induced DPN mouse model. Daily intraplantar (i.pl.) injections of β-caryophyllene (BCP) (27 µg) significantly alleviated mechanical allodynia in diabetic mice compared to vehicle-treated diabetic controls on days 7, 14, and 21. In contrast, topical analgesic cream containing capsaicin 0.075% showed a significant effect only on day 21. CB_2_ receptor antagonist AM630 pretreatment abolished this effect, while CB_1_ receptor antagonist AM251 had no significant impact. Non-diabetic mice maintained higher thresholds throughout the study. Data are presented as mean ± SEM (*n* = 8 per group). Statistical analysis: two-way ANOVA with Tukey’s post-hoc test. ns, not significant, * *p* < 0.05, *** *p* < 0.001 compared to diabetic controls; ns indicates no significant difference compared to diabetic controls; ~~~ *p* < 0.001 compared to BCP (27 µg)-treated diabetic mice.

The chronic effects of BCP were evaluated through daily i.pl. injections of 27 µg BCP over 21 days, beginning 14 days post-STZ injection. Mechanical allodynia was assessed 5 min after each BCP administration on days 7, 14, and 21 (Figure 5). Chronic BCP treatment significantly alleviated mechanical allodynia, with paw withdrawal thresholds consistently higher than those of vehicle-treated diabetic controls on all evaluated days (*p* < 0.001). These results indicate sustained anti-allodynic effects throughout the 21-day treatment period. Mechanistic studies confirmed that pretreatment with the CB_2_ antagonist AM630 effectively abolished BCP’s anti-allodynic effects, verifying the involvement of CB_2_-mediated activity. Conversely, the CB_1_ antagonist AM251 had no significant impact, further supporting the specificity of CB_2_ receptor activation.

As a clinical comparator, mice received a single daily dose of topical analgesic cream containing capsaicin 0.075%. No improvement in mechanical allodynia was observed on days 7 and 14. However, a significant reduction in mechanical allodynia was noted on day 21 (*p* < 0.01). In conclusion, daily i.pl. injections of 27 µg BCP provide sustained relief from diabetic neuropathic pain and demonstrate superior efficacy over topical capsaicin cream (0.075%). These findings position BCP as a promising therapeutic agent for the effective management of diabetic peripheral neuropathy.

### 2.4. Chronic Topical BCP Administration Modulates Pro-Inflammatory Cytokine Levels in STZ-Induced DPN Mouse Model

**Figure 6:**
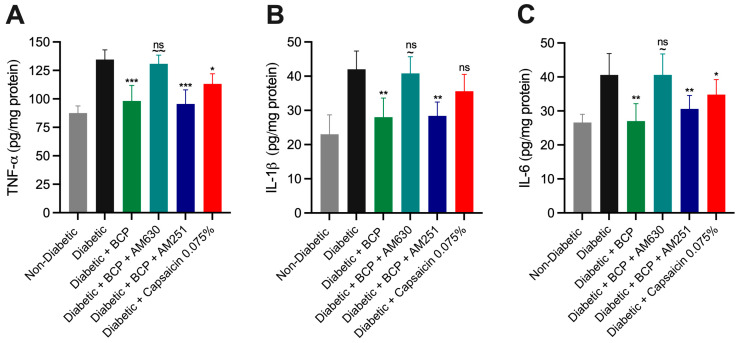
Topical intraplantar (i.pl.) BCP administration modulates pro-inflammatory cytokines in hind paw skin tissues of STZ-induced DPN mouse model. (**A**) Tumor necrosis factor-alpha (TNF-α), (**B**) interleukin-1β (IL-1β), and (**C**) interleukin-6 (IL-6) levels were assessed in hind paw tissues of non-diabetic and diabetic mice using ELISA. Diabetic mice exhibited significantly elevated cytokine levels compared to non-diabetic controls. Topical i.pl. treatment with BCP (27 µg) significantly reduced these cytokine levels. Pretreatment with the CB_2_ antagonist AM630 abolished the anti-inflammatory effects of BCP, while the CB1 antagonist AM251 had no significant impact. Topical capsaicin 0.075% significantly reduced TNF-α and IL-6 levels but did not affect IL-1β. Data are expressed as mean ± SEM (*n* = 8 per group). Statistical analysis: one-way ANOVA with Tukey’s post-hoc test. ns, not significant, * *p* < 0.05, ** *p* < 0.01, *** *p* < 0.001 compared to diabetic controls; ns indicates no significant difference compared to diabetic controls; ~~ *p* < 0.01, ~ *p* < 0.05 compared to BCP (27 µg)-treated diabetic mice.

Inflammation is a key factor in the pathogenesis of DPN, with pro-inflammatory cytokines significantly contributing to neuropathic pain and tissue damage. To evaluate the anti-inflammatory potential of chronic topical BCP treatment, we measured TNF-α IL-1β) and IL-6 levels in hind paw skin tissues using ELISA (Figure 6A–C). Diabetic mice exhibited markedly elevated cytokine levels compared to non-diabetic controls (*p* < 0.001). Daily i.pl. administration of BCP (27 µg for 21 days) significantly reduced these elevated cytokine levels (*p* < 0.01), demonstrating its potent anti-inflammatory effects. The CB_2_ receptor antagonist AM630 effectively reversed the cytokine-lowering effects of BCP (*p* < 0.01), confirming the involvement of CB_2_ receptor activation. In contrast, the CB_1_ receptor antagonist AM251 had no significant impact.

Topical capsaicin cream (0.075%) reduced TNF-α and IL-6 levels (*p* < 0.05) but was less effective than BCP (27 µg). Notably, capsaicin cream had no significant effect on IL-1β levels. These results highlight BCP’s ability to selectively modulate pro-inflammatory cytokines involved in DPN progression. Its efficacy, primarily mediated through CB_2_ receptor activation, underscores its potential to mitigate neuroinflammation in hind paw skin tissues and improve outcomes in diabetic neuropathy.

### 2.5. Effect of BCP on Oxidative Stress Biomarkers in Hind Paw Skin Tissues of STZ-Induced DPN Mice

**Figure 7:**
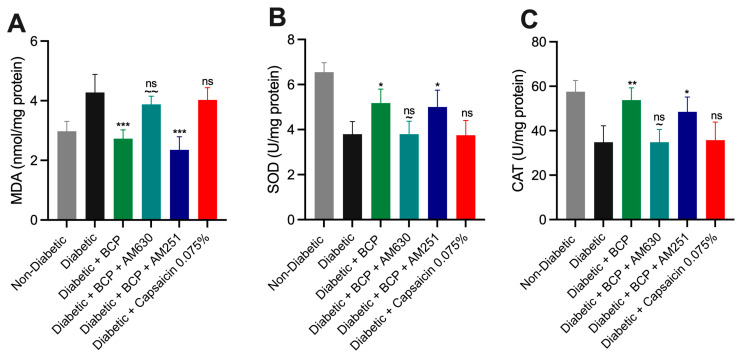
Effect of topical β-Caryophyllene (BCP) administration on oxidative stress biomarkers in hind paw skin tissues of STZ-induced DPN mouse model. (**A**) malondialdehyde (MDA) levels, (**B**) superoxide dismutase (SOD) activity, and (**C**) catalase (CAT) activity were measured in skin homogenates from hind paw tissues. BCP treatment significantly restored SOD and CAT activities while reducing MDA levels in diabetic mice. These effects were reversed by pretreatment with AM630 (CB_2_ receptor antagonist), confirming CB_2_-mediated mechanisms. AM251 (CB_1_ receptor antagonist) had no significant impact. Topical capsaicin 0.075% did not alter oxidative stress markers. Statistical analysis: one-way ANOVA with Tukey’s post-hoc test. *** *p* < 0.001, ** *p* < 0.01, * *p* < 0.05 compared to diabetic controls; ns indicates no significant difference compared to diabetic controls; ~~ *p* < 0.01, ~ *p* < 0.05 compared to BCP (27 µg)-treated diabetic mice. Data are presented as mean ± SEM (*n* = 8 per group).

Oxidative stress plays a critical role in tissue damage and the progression of DPN, as evidenced by increased lipid peroxidation and impaired antioxidant defenses. The effect of BCP on oxidative stress in hind paw skin tissues was assessed by measuring MDA, a marker of lipid peroxidation, and the activities of antioxidant enzymes SOD and CAT (Figure 7A–C). STZ-induced diabetic mice exhibited a significant increase in MDA levels (*p* < 0.001) alongside notable reductions in SOD (*p* < 0.05) and CAT (*p* < 0.01) activities compared to non-diabetic controls, indicating heightened oxidative stress. Chronic topical BCP administration (27 µg for 21 days) significantly reduced MDA levels while restoring SOD and CAT activities (*p* < 0.05) relative to vehicle-treated diabetic mice. The involvement of CB_2_ receptors in mediating BCP’s antioxidative effects was confirmed by pretreatment with the CB_2_ receptor antagonist AM630, which significantly reversed these effects (*p* < 0.05). In contrast, the CB_1_ receptor antagonist AM251 had no significant impact, underscoring the specificity of CB_2_ receptor activation. Topical capsaicin cream (0.075%) did not influence oxidative stress biomarkers, showing no effect on MDA levels or SOD and CAT activities. These findings demonstrate that topical BCP treatment effectively mitigates oxidative damage in hind paw skin tissues of STZ-induced DPN mice, primarily through CB_2_ receptor activation, highlighting its potential as a targeted therapeutic strategy for DPN.

## 3. Discussion

This study provides robust evidence supporting the therapeutic value of topical BCP administration in alleviating mechanical allodynia in an STZ-induced DPN mouse model. The findings demonstrate that BCP exerts dose-dependent and sustained efficacy through chronic administration, with its action explicitly mediated by CB_2_ receptors in peripheral tissues. Importantly, BCP’s effects were found to be independent of CB_1_ receptor activation and central mechanisms involving the sciatic nerve, further reinforcing its localized mechanism of action. These insights highlight BCP as a promising candidate for managing DPN and addressing the limitations of current therapies, which are often accompanied by undesirable side effects.

STZ-induced diabetic mice exhibited persistent hyperglycemia (>250 mg/dL) and significant weight loss, which are hallmark features of well-established diabetes models [28]. These systemic metabolic disturbances confirm the effective induction of diabetes. Notably, the topical administration of BCP, regardless of the dose, did not alter blood glucose levels or body weight in diabetic mice. This observation aligns with prior studies indicating that BCP’s therapeutic effects are independent of systemic metabolic regulation [25]. The ability of BCP to act locally without affecting systemic glucose metabolism or body weight provides a key advantage over traditional DPN therapies, which often have systemic side effects like sedation or gastrointestinal disturbances [9].

Pharmacological validation confirmed that BCP’s analgesic effects are mediated through CB_2_ receptor activation. Pretreatment with the CB_2_ antagonist AM630 abolished BCP’s anti-allodynic effect, while the CB_1_ antagonist AM251 had no impact, confirming BCP’s peripheral and CB_2_-specific mechanism of action. Although CB_2_ receptor expression in hind paw tissues was not directly assessed due to limited sample availability, functional evidence supports its involvement. Prior studies have demonstrated CB_2_ expression in peripheral tissues—including nociceptive neurons and immune cells—particularly under neuropathic or inflammatory conditions [29,30,31]. These receptors mediate antinociceptive effects through modulation of peripheral inflammation and, under some conditions, central microglial activation [26,31,32,33]. Future studies will include molecular confirmation using Western blot or immunohistochemistry.

The intraplantar route was chosen to evaluate the direct peripheral effects of BCP at the site of nociceptive sensitization in DPN. While DPN is systemic, localized treatment allows site-specific targeting and may reduce systemic side effects. This approach also enabled precise pharmacological validation of CB_2_ receptor involvement using selective antagonists. Although systemic BCP has shown analgesic efficacy in previous studies, our study provides proof-of-concept for localized BCP therapy, with potential for development into topical or transdermal formulations for targeted DPN management.

Our findings align with previous studies showing that localized BCP injections alleviate mechanical allodynia via CB_2_ receptor-mediated mechanisms in neuropathic models like partial sciatic nerve ligation [24]. Systemic BCP, alone or with cannabidiol, has also been effective in STZ-induced diabetic rodents, confirming its broader analgesic potential [19,25]. These results highlight BCP’s therapeutic versatility across both delivery routes. While our study focused on topical administration, future comparisons with systemic delivery are needed to clarify relative benefits. Significantly, targeting peripheral CB_2_ receptors may help avoid side effects associated with CB_1_ activation or conventional treatments [9]. Comparative studies are warranted to fully explore the differential benefits of topicalized versus systemic BCP therapy. While previous studies have demonstrated systemic BCP efficacy in neuropathic pain models, our study specifically validated the peripheral CB_2_ receptor-mediated effects of topical BCP administration in DPN. Future investigations comparing systemic versus topical delivery strategies are essential to determine the optimal approach for maximizing therapeutic efficacy while minimizing systemic side effects.

In addition to its analgesic effects, BCP’s capacity to attenuate pro-inflammatory cytokines, including TNF-α, IL-1β, and IL-6, further underscores its significant therapeutic potential in addressing the inflammatory underpinnings of DPN. These cytokines are critical mediators of neuroinflammation and nociceptor sensitization, which drive the pathophysiology of neuropathic pain in the DPN [7,8]. Several studies support the anti-inflammatory role of CB_2_ receptor agonists, emphasizing their ability to suppress neuroinflammatory cascades and attenuate nociceptor excitability in neuropathic pain models [14,19,25,34,35]. For instance, Aguilar-Ávila et al. (2019) demonstrated that chronic systemic administration of BCP in STZ-induced diabetic mice significantly reduced elevated levels of Substance P and cytokines, including TNF-α, IL-1β, and IL-6, in blood serum, aligning with BCP’s well-documented anti-inflammatory properties [25]. Similarly, Khan et al. (2024) reported that chronic intraperitoneal treatment with a combination of BCP and cannabidiol significantly decreased NFκB, IL-1β, and IL-18 levels in STZ-induced DPN rats [19]. These findings reinforce the efficacy of BCP in modulating key inflammatory mediators in diabetic neuropathic pain models. In our study, topical administration of BCP significantly reduced inflammation in hind paw skin tissues, as evidenced by decreased TNF-α, IL-1β, and IL-6 levels. The CB_2_ receptor antagonist AM630 completely abolished BCP’s anti-inflammatory effects, confirming that these actions are mediated via CB_2_ receptor activation.

Oxidative stress is another key contributor to DPN, arising from chronic hyperglycemia-induced ROS that overwhelm antioxidant defenses [7,8]. In this study, STZ-induced diabetic mice exhibited elevated MDA levels and reduced activities of antioxidant enzymes SOD and CAT in hind paw skin tissues, reflecting oxidative imbalance and nerve dysfunction. Topical BCP administration significantly reduced MDA levels and restored SOD and CAT activities, effects that were abolished by the CB_2_ antagonist AM630 but unaffected by the CB1 antagonist AM251. These results confirm CB_2_ receptor activation as the mechanism behind BCP’s antioxidative effects. Consistent with prior studies, BCP mitigates oxidative stress in neuropathic pain models by enhancing antioxidant activity and reducing ROS production [14,15,18]. CB_2_ activation provides a dual-action approach, targeting oxidative and inflammatory pathways, offering a comprehensive strategy for managing DPN [13,36]. BCP’s topical administration provides a targeted and efficient approach to DPN management, reducing oxidative damage while avoiding systemic side effects.

In addition to CB_2_ receptor activation, BCP may engage complementary molecular pathways that contribute to its multifaceted analgesic, anti-inflammatory, and antioxidant effects in DPN. BCP is a known peroxisome proliferator-activated receptor gamma (PPAR-γ) agonist, contributing to anti-inflammatory and metabolic regulation [36], and it activates the Nrf2/HO-1 antioxidant pathway, enhancing cellular resilience against oxidative stress [37]. Additionally, BCP may influence adenosine signaling, particularly through A2A receptor modulation, contributing to its neuroprotective and anti-inflammatory actions [38]. While BCP does not directly activate the transient receptor potential vanilloid 1 (TRPV1) channel, emerging evidence suggests it may regulate TRPV1 expression under pathological conditions. For example, Serra et al. (2022) showed that BCP administration before cerebral hypoperfusion/reperfusion injury restored TRPV1 levels in the frontal cortex of rats and modulated the BDNF-trkB signaling system [39]. These findings suggest BCP plays a role in mitigating neuroinflammation by influencing TRPV1 and neurotrophic pathways. These complementary mechanisms may work in concert with CB_2_ receptor activation to produce a more comprehensive therapeutic effect in managing DPN by targeting nociceptive sensitization, inflammation, and oxidative damage.

Capsaicin 0.075% cream was included as a clinical comparator rather than a control, as it represents an FDA-approved topical treatment for peripheral neuropathic pain through transient receptor potential vanilloid 1 (TRPV1)-mediated nociceptor desensitization. However, in our study, the group treated with topical capsaicin cream (0.075%) demonstrated limited efficacy, while the cream significantly reduced TNF-α and IL-6 levels, as reported previously [40,41]. However, capsaicin cream (0.075%) had no impact on IL-1β levels, nor did it improve oxidative stress markers (MDA, SOD, and CAT). This aligns with the existing literature suggesting that capsaicin’s primary mechanism of action is through desensitization of nociceptive neurons rather than modulation of inflammatory or oxidative stress pathways [42]. Although capsaicin creams are clinically available for neuropathic pain, including DPN, their prolonged application requirement and limited scope in addressing underlying neuroinflammatory and oxidative stress mechanisms restrict their effectiveness. Studies have indicated that higher concentrations, such as 0.75% capsaicin, might be necessary to achieve significant analgesic effects in animal models [27]. These limitations further emphasize the superior therapeutic scope of BCP.

It is important to note that while capsaicin 0.075% cream was used as a clinical comparator, its mechanism of action—TRPV1-mediated nociceptor desensitization—and method of administration differ fundamentally from those of BCP. Our study’s primary focus was to elucidate the peripheral CB_2_ receptor-mediated analgesic, anti-inflammatory, and antioxidative effects of BCP, rather than to perform a direct pharmacological comparison between the two agents. The inclusion of capsaicin highlights the limited efficacy of current topical treatments and underscores the therapeutic promise of targeting CB_2_ receptors for more comprehensive management of DPN.

Topical BCP administration offers a targeted approach to managing DPN, providing pain relief with minimal systemic exposure. Unlike systemic treatments like pregabalin and duloxetine, BCP’s CB_2_-specific mechanism avoids common side effects such as sedation and gastrointestinal discomfort [2,9,10]. While topical treatments like lidocaine patches offer only temporary relief, BCP’s dual anti-inflammatory and antioxidative actions address the underlying neuroinflammation and oxidative stress in DPN [13,36]. Recent studies further support CB_2_ receptor modulation as an effective strategy for managing DPN [19,25]. However, clinical trials are essential to confirm BCP’s safety, optimize its dosage, and evaluate its integration with current DPN treatment paradigms.

Recent studies suggest that developing alternative delivery methods for BCP—such as topical formulations and nanoencapsulation—could significantly enhance its clinical utility in managing DPN. Topical BCP-based creams, gels, or transdermal patches could offer targeted, non-invasive pain relief with fewer systemic side effects, making them attractive options for long-term use. Additionally, nanoencapsulation strategies have been shown to improve BCP’s bioavailability, chemical stability, and controlled release, thereby increasing therapeutic efficacy while minimizing systemic exposure [43,44]. Preclinical research using BCP-loaded nanoparticles has demonstrated promising results for localized neuropathic pain management [45,46]. Together, these innovations support the development of clinically viable BCP formulations that could enhance treatment adherence, extend the duration of action, and broaden therapeutic outcomes for patients with DPN.

## 4. Materials and Methods

### 4.1. Drugs and Reagents

β-Caryophyllene (BCP; Catalog No: HY-N1415, MedChemExpress, Deer Park, NJ, USA) was prepared in a vehicle consisting of 90% saline, 5% dimethyl sulfoxide (DMSO; Sigma-Aldrich, Burlington, MA, USA), and 5% Kolliphor (Sigma-Aldrich, Burlington, MA, USA). For topical delivery, BCP was administered via intraplantar (i.pl.) injections into the plantar surface of the hind paw. AM251 (Catalog No: HY-15443, MedChemExpress, Deep Park, NJ, USA), a selective CB_1_ receptor antagonist, and AM630 (Catalog No: A3168, APExBio, Houston, TX, USA), a selective CB_2_ receptor antagonist, were prepared in the exact vehicle. These antagonists were administered i.pl. 30 min before BCP injection to ensure effective receptor blockade. Intraplantar injections were administered with a standardized volume of 20 µL per site to ensure consistent dosing, utilizing a microsyringe Hamilton Company (Bonaduz, Switzerland) equipped with a 30-gauge needle. Streptozotocin (STZ; Catalog No: S0130, Sigma-Aldrich (Burlington, MA, USA) was freshly dissolved in 0.1 M citrate buffer (pH 4.5) immediately before administration to ensure its stability. STZ was administered intraperitoneally (i.p.) following established protocols [28]. All drugs and reagents were stored at −20 °C and thawed to room temperature before use.

### 4.2. Ethics Statement

The research received ethical approval from the Faculty of Pharmacy Ethical Committee at King Abdulaziz University (Protocol No: PH-112-40). All experimental procedures strictly adhered to the National Institutes of Health guidelines for the Care and Use of Laboratory Animals [47]. Humane endpoints were strictly monitored to minimize animal suffering. Criteria for euthanasia included severe distress or health complications. Animals meeting these criteria were humanely euthanized following ethical guidelines [48].

### 4.3. Experimental Diabetes Induction

Swiss Webster (SWR) male mice (20–25 g) were housed under standard laboratory conditions at the Faculty of Pharmacy, King Abdulaziz University’s animal care facility. The animals were kept under controlled conditions at a temperature of 25 °C, with a 12-h light/dark cycle, and were provided unrestricted access to food and water. The experimental timeline is outlined in Figure 1. A total of 70 mice were used in this study. An STZ-induced DPN mouse model was established by inducing diabetes via i.p. injections of STZ (90 mg/kg) administered on two consecutive days, following established protocols [28]. Mice injected with 0.1 M citrate buffer (pH 4.5) alone were non-diabetic controls. Body weight and fasting blood glucose levels were monitored weekly throughout the study to track diabetes progression. Blood glucose levels were measured from tail vein samples using a digital glucometer (Accu-Chek Instant; Roche Diagnostics, Basel, Basel-Stadt, Switzerland) from tail vein samples. Mice were considered diabetic if their fasting blood glucose levels exceeded 250 mg/dL two weeks following the final STZ injection. Animals that did not meet this criterion were excluded from the study. Mice were monitored regularly for signs of distress, and humane endpoints were defined based on health status and body condition scores.

Fourteen days after STZ injection, hyperglycemic mice were randomly allocated into eight experimental groups (*n* = 8 per group) using a randomized block design. At this stage, diabetic mice exhibited clinical features consistent with DPN, including hyperglycemia and mechanical hypersensitivity. The experimental groups included a vehicle control group (diabetic) that received i.pl. injections of the vehicle. In this study, the vehicle control group received intraplantar injections of the BCP vehicle (90% saline, 5% DMSO, 5% Kolliphor), which was required for BCP solubilization. A separate saline-only control group was not included as the vehicle was predominantly saline-based. Three BCP treatment groups received intraplantar (i.pl.) injections of BCP at doses of 9, 18, or 27 µg, respectively. These doses were selected based on previous studies demonstrating the efficacy of BCP in neuropathic pain models [24]. Two combination treatment groups were administered 27 µg BCP i.pl. along with either 12 µg AM251 (CB_1_ receptor antagonist) or 4 µg AM630 (CB_2_ receptor antagonist) [24]. A control group received topical analgesic cream with capsaicin 0.075% (Zostrix^®^ HP). This randomization ensured equal distribution of treatment conditions and maintained experimental consistency. Non-diabetic mice were i.pl. injected within the vehicle alone. BCP treatments were administered daily for 21 days, starting 14 days post-STZ injection.

Twenty-four hours after the final behavioral and treatment assessments, the mice were euthanized via an intraperitoneal overdose of pentobarbital (100 mg/kg, i.p.). Skin samples from the plantar region of the hind paws were quickly collected, immediately snap-frozen in liquid nitrogen, and preserved at −80 °C for later analysis.

### 4.4. Assessment of Mechanical Allodynia

Mechanical allodynia was evaluated using manual von Frey filaments (Ugo Basile, Gemonio, Varese, Italy) following established protocols [49,50]. Each mouse was placed individually in a transparent Plexiglas chamber on a wire mesh platform and allowed to acclimate for 30 min to minimize stress and ensure reliable behavioral responses. A series of calibrated von Frey filaments (0.16–8 g) were applied perpendicularly to the plantar surface of the hind paw for 6 s. The paw withdrawal threshold was determined using the up-down method, a standard and reliable approach for evaluating mechanical nociception. Each hind paw was tested three times in random order, and the mean withdrawal threshold was calculated. Testing was conducted by a blinded observer to minimize bias and ensure objective results. Observers were trained in the methodology to standardize procedures, and the testing sequence (e.g., left or right paw first) was randomized. Mice with abnormal baseline responses were excluded from further analysis. These measures ensured reliable and reproducible assessments of mechanical allodynia.

### 4.5. Enzyme-Linked Immunosorbent Assay (ELISA)

ELISA was employed to quantify TNF-α, IL-6, and IL-1β levels in skin tissue homogenates from the plantar region of the hind paws. At the end of the experiment, 6 mm skin punches were collected, thoroughly rinsed in ice-cold phosphate-buffered saline (PBS, pH 7.4) to remove excess blood, and accurately weighed. The tissues were minced and homogenized in PBS (tissue weight (g):PBS (mL) = 1:9) using a glass homogenizer on ice to maintain protein integrity. The resulting homogenates were centrifuged at 12,000 RPM for 15 min at 4 °C, and the supernatants were carefully collected and stored at −80 °C until analysis, avoiding freeze/thaw cycles. The concentrations of TNF-α (Cat. No: E0117Mo), IL-6 (Cat. No: E0049Mo), and IL-1β (Cat. No: E0119Mo) were measured using commercially available ELISA kits (Bioassay Technology Laboratory, Beijing, China) according to the manufacturer’s protocols. Optical density was determined at 450 nm using a SpectraMax^®^ M2 microplate reader (San Jose, CA, USA) and analyzed with SoftMax® Pro Software, version 7.1 (Molecular Devices, San Jose, CA, USA) [51]. The total protein concentration in each sample was measured using the bicinchoninic acid (BCA) protein assay (Thermo Scientific, Waltham, MA, USA) following the manufacturer’s instructions. All cytokine concentrations were normalized to total protein content and expressed as pg/mg protein.

### 4.6. Assessment of Oxidative Stress Biomarkers in Hind Paw Skin Tissues

Oxidative stress biomarkers were assessed in homogenized skin tissue samples from the hind paws to determine the effects of STZ-induced neuropathy. Malondialdehyde (MDA) levels (Catalog No: MD2529, Biodiagnostic, Cairo, Egypt) were measured to indicate lipid peroxidation. Additionally, the activities of essential antioxidant enzymes, catalase (CAT) and superoxide dismutase (SOD) (Catalog No: CA2517 and SD2521, Biodiagnostic, Cairo, Egypt), were evaluated using commercial assay kits according to the manufacturer’s instructions. Total protein content was quantified using the BCA protein assay (Thermo Scientific, Waltham, MA, USA). MDA levels were expressed as nmol/mg protein, while CAT and SOD activities were expressed as U/mg protein.

### 4.7. Statistical Analyses

Data are expressed as mean ± standard error of the mean (SEM). Data were tested for normality using the Shapiro–Wilk test. All datasets conformed to a normal distribution, allowing for the use of parametric tests (ANOVA and Tukey’s post hoc). Statistical analysis was performed using one-way or two-way analysis of variance (ANOVA) to evaluate the influence of treatment and time. Tukey’s post-hoc test was applied for comparisons across multiple groups, while Bonferroni correction was used for specific pairwise comparisons. A *p*-value < 0.05 was considered statistically significant. All statistical analyses were conducted using GraphPad Prism version X.

## 5. Conclusions

This study demonstrates that topical BCP administration significantly alleviates mechanical allodynia in an STZ-induced DPN model. Its therapeutic effects are mediated primarily through CB_2_ receptor activation in peripheral tissues, independent of central mechanisms or CB_1_ receptor involvement. BCP also effectively reduces pro-inflammatory cytokines and oxidative stress markers, supporting its dual anti-inflammatory and antioxidant action. Compared to standard treatments, BCP offers a targeted, well-tolerated, and mechanistically comprehensive approach. With its multifaceted pharmacological profile and promising safety, BCP emerges as a strong candidate for long-term DPN management, pending validation in clinical studies.

## Data Availability

The data supporting this study’s findings are available from the corresponding author upon reasonable request.
